# Surgical treatment of carpometacarpal thumb arthritis with trapeziectomy and intra-tendon (FCR) suspension with one-loop APL: comparative cohort study

**DOI:** 10.1186/s12891-023-06420-y

**Published:** 2023-04-25

**Authors:** Marco Passiatore, Giuseppe Taccardo, Vitale Cilli, Giuseppe Rovere, Francesco Liuzza, Lucia Pannuto, Rocco De Vitis

**Affiliations:** 1 Department of Bone and Joint Surgery, Spedali Civili, 25121 Brescia, Italy; 2 Department of Orthopaedics, Orthopaedic Institute, Fondazione Policlinico Universitario A. Gemelli IRCCS - Università Cattolica del Sacro Cuore, Largo Agostino Gemelli N°8, 00168 Rome, Italy; 3 Hand Surgery Unit, CHIREC Site Delta, 1160 Bruxelles, Belgium; 4 Queen Victoria Hospital NHS Foundation Trust, East Grinstead, RH19 3DZ UK

**Keywords:** Carpometacarpal thumb arthritis, Trapeziectomy, Tendons, Loop

## Abstract

**Background:**

One of the current choices of treatment for Trapeziometacarpal (TMC) joint arthritis is trapeziectomy with ligament reconstruction and tendon interposition arthroplasty. The Ceruso’s technique consists of complete trapezial excision and abductor pollicis longus (APL) tendon suspension. The APL tendon is tied to the flexor carpi radialis (FCR) tendon with two loops, one around it and one inside, and then used as interposition tissue. The purpose of the present study was to compare two different techniques of a trapeziectomy with ligament reconstruction and tendon interposition arthroplasty using the Abductor Pollicis Longus (APL) tendon, which is only Once Looped Around (OLA) versus Once Looped Inside (OLI) the Flexor Carpi Radialis (FCR) tendon.

**Methods:**

A single-center, retrospective study (Level of evidence: III) has been conducted on sixty-seven patients older than 55 years (33 OLI, 35 OLA), assessing clinical outcomes for at least 2 years of post-surgery follow-up. The outcomes were to assess and compare surgical outcomes comparing the two groups, in terms of subjective and objective evaluation for both groups at the last follow-up (primary outcome), and at the intermediate follow-ups (three and six months). Complications were also assessed.

**Results:**

The authors found an improvement in pain, range of motion, and function, with equivalent results for both techniques. No subsidence was observed. FCR tendinitis was significantly reduced with OLI, as well as the need of post-operative physiotherapy.

**Conclusions:**

The one-loop technique allows for reduced surgical exposure, providing excellent suspension and clinical outcomes. Intra FCR loop should be preferred to improve post-surgical recovery.

**Level of evidence:**

Level III study. This is a retrospective cohort study (written according to STROBE guidelines).

## Background

Osteoarthritis of the trapeziometacarpal joint of the thumb is a painful and debilitating condition of the hand with a reported age-adjusted prevalence of 7% in men and 15% in women [1]. It typically presents in postmenopausal females, and patients often complain of pain at the base of the thumb during activities which require opposition or pinch grip [2]. Conservative treatment involves activity modification, non-steroidal anti-inflammatory drugs (NSAIDS), splinting, and corticosteroid injections [2]. When these fail, surgical treatment should be considered to provide pain relief and restoring thumb motion and strength [2].

**Fig. 1:**
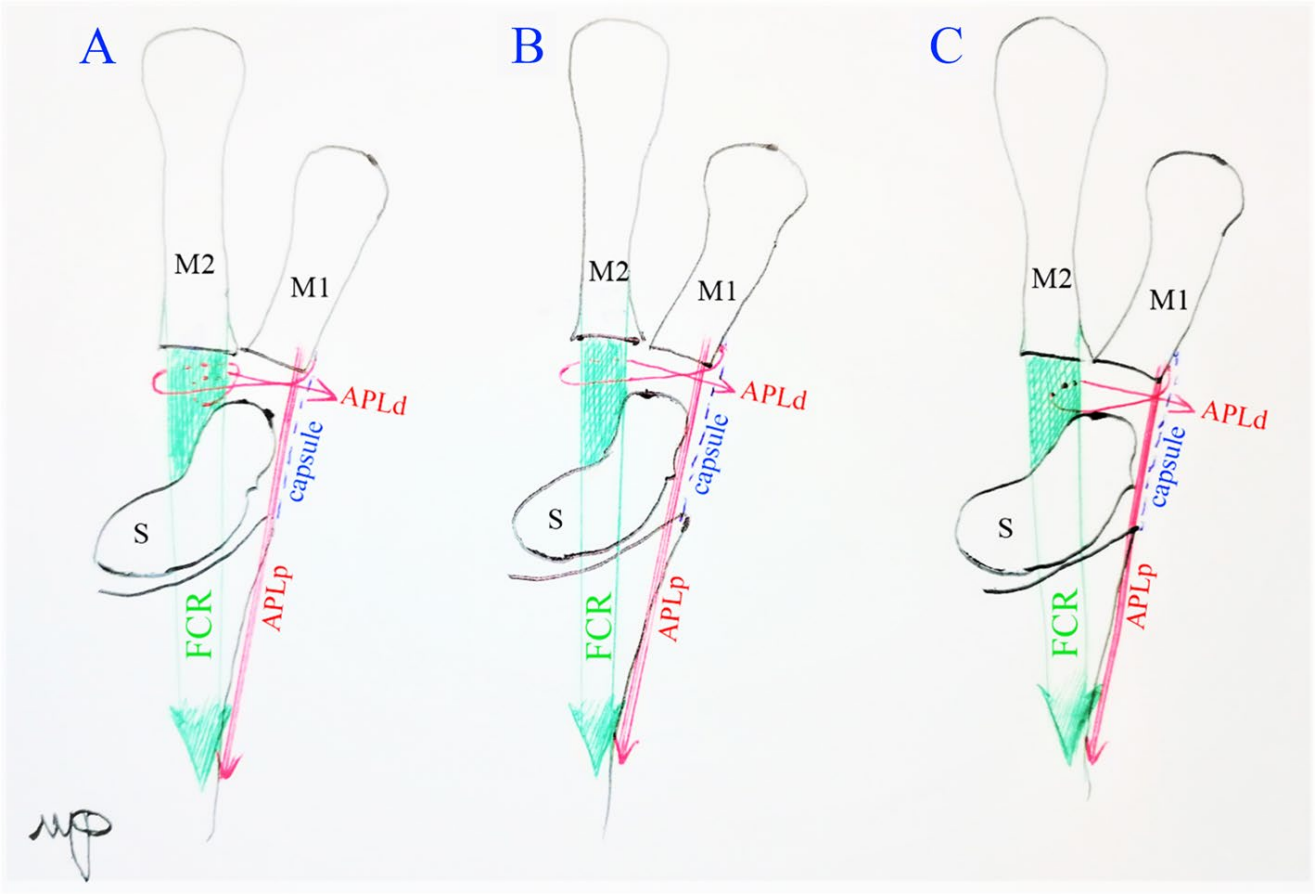
**A** Ceruso’s technique. **B** Catalano’s modification, called the OLA (once-looped around) technique. **C** Once-looped intratendineous” (OLI) technique. M1: first metacarpal bone. M2: second metacarpal bone. S: scaphoid. FCR: flexor carpi radialis. APLp: palmar slip of abductor pollicis longus. APLd: dorsal slip of abductor pollicis longus. The APLd is sutured on the capsule after the interposition, and the capsule is closed

In advanced stages, (stage 3 or 4 according to Dell et al.), [2–4] reconstructive procedures are needed due to loss of articular cartilage, and can be grouped into four main categories: (1) trapezial excision [5]; (2) trapeziectomy with soft tissue arthroplasty [6]; (3) arthrodesis [7]; (4) total joint replacement arthroplasty (prosthetic arthroplasty) [8]. Nowadays, the most performed surgical procedure is known as Ligament Reconstruction and Tendon Interposition procedure or LRTI, which was first described in 1986 [6, 9]. According to the first description of the LRTI, it involves thumb ligament reconstruction with half of the width of the FCR tendon routed through the base of the thumb metacarpal, while the trapeziectomy void is filled with the remaining length of the tendon. Various modifications of this so-called suspension arthroplasty technique have been proposed over the years, designed to improve thumb stability and prevent metacarpal subsidence. Hence, the choice of tendon used to the make the suspension and fill trapeziectomy void can differ [2, 6, 10, 11]. The technique described by Weilby is one of the most commonly used [12]. It avoids the use of bony tunnels by simply weaving a slip of FCR around the APL and the remaining FCR in a figure of eight fashion [12]. Ceruso’s modification of Weilby’s technique consists of first looping the APL around the FCR and then inside the FCR [13–15]. The rest of the slip is used to fill the trapeziectomy void (Fig. 1A) [16].

In our center, for several years we routinely performed a simplified version of this technique with a single-loop passage around the FCR (Catalano’s modification), hereinafter called the OLA (one-loop around) technique (Fig. 1B). Our belief was that one loop was enough to maintain the suspension. In this way, we could take a shorter APL slip with a smaller exposure and minor scar. According to the literature about Ceruso’s original technique, this method resulted in excellent outcomes throughout the years despite several cases of FCR tendinitis as main complication, probably due to FCR synovitis caused by loop passage around the FCR [15].

Therefore in 2015, we made a further adjustment to the technique. The external weaving of the APL tendon around the FRC tendon was avoided, and the slip of the APL was passed through the FCR tendon and fixed without additional maneuvers. We have called this further modification “one-loop inside” (OLI) technique (Fig. 1C).

The rationale of the OLI technique is to reduce the stress on the tendon sheath of the FCR caused by the APL loop. In our opinion, the FCR tendon is a sufficiently large tendon that half of it could be sufficient for optimal tendon suspension of the thumb, without loss of tension and providing a gliding pulley for the APL tendon.

Our study is aimed at assessing the surgical outcomes and complication rates of these two techniques, OLA and OLI.

## Materials and methods

The present investigation consists in a retrospective analysis on the institutional database of a single Hospital. This manuscript was reported following the STROBE guidelines. The approval of the institutional review board was obtained. The study was performed in accordance with the Helsinki Convention. An ethical approval was not requested for this retrospective analysis. All patients affected by trapeziometacarpal (TMC) joint arthritis who underwent surgery from January 2015 to April 2018 were considered. A written consent was obtained from all patients before surgery.

A retrospective study has been conducted on 201 consecutive patients affected by TMC joint arthritis who have been treated at our center between January 2015 and April 2018. The inclusion criteria were:

- Patients older than 55 years;
- Stage III-IV disease of Eaton and Littler [4].
- At least 2 years of post-surgery follow-up.

The exclusion criteria were the presence of hand polyarthritis (confirmed by the preoperative X-ray), rheumatic diseases, neuromuscular disease, scaphoid-trapezial-trapezoidal arthritis, previous corticosteroid injection in TMC joint and the simultaneous presence of other hand diseases (such as carpal tunnel syndrome, tendinitis, etc.) with or without indication to surgery. Patients with severe metacarpo-phalangeal (MP) hyperextension (> 20°) were also excluded from the study. In the event of hyperuricemia, patients were prescribed treatment and then required to repeat blood tests, to confirm the normalization before surgery. Furthermore, patients with a follow-up period shorter than 2 years, or who had undergone reoperation, or who had suffered fractures to the hand and wrist before the last follow-up were excluded from the study. Finally, patients with incomplete pre- or post-operative data set were excluded from the study.

Pre-operative dataset included general demographic, clinical history, Charlson Comorbidity Index (CCI) score, X-rays of both hands (anterior–posterior, lateral and oblique view), thumb opposition score of Kapandji, strength measured through dynamometers (grip, lateral pinch and tip pinch strength), Michigan Hand Outcome (MHQ) [17] and DASH questionnaires [18], pain. Post-operative dataset included X-rays of the operated hand (anterior–posterior, lateral and oblique view) for the evaluation of suspension loss, thumb opposition score of Kapandji, strength, Michigan Hand Outcome (MHQ) and DASH questionnaires, pain, general satisfaction. Post-operative complications (in particular FCR tendinitis), need for physiotherapy.

Given the retrospective evaluation no randomization was performed and eligible patients were divided into two groups as below considering the description of the surgery:

- Group A: patients operated on with the OLA technique;
- Group B: patients who underwent OLI technique.

All patients were operated on by three authors and signed an informed consent before surgery.

### Surgical technique

Surgery is performed under peripheral anesthesia and transient ischemia (pneumatic cuff) by two expert hand surgeons (R.D.V. and G.T.). The skin is incised longitudinally (approximately 40–45 mm) along the course of the APL, centered on the TMC joint. The sensory branches of the radial nerve and the radial artery are identified and protected, the joint capsule is Y-incised to preserve a large distal triangular flap, the basis of which is inserted on the first metacarpal bone. Trapeziectomy is performed. The FCR tendon is exposed at the bottom of the surgical field. The APL tendon is commonly composed of several distinct subunits; the most dorsal subunit should be used to perform the arthroplasty and tendon interposition, after ensuring that it inserts at the base of the first metacarpal bone. The selected portion is then isolated and dissected proximally about 6 cm from its distal insertion. The skin at that point is very elastic. Excellent exposure is achieved by lifting the proximal end of the skin incision. Furthermore, the tendon is harvested without opening the pulley of the first dorsal compartment.

**Fig. 2:**
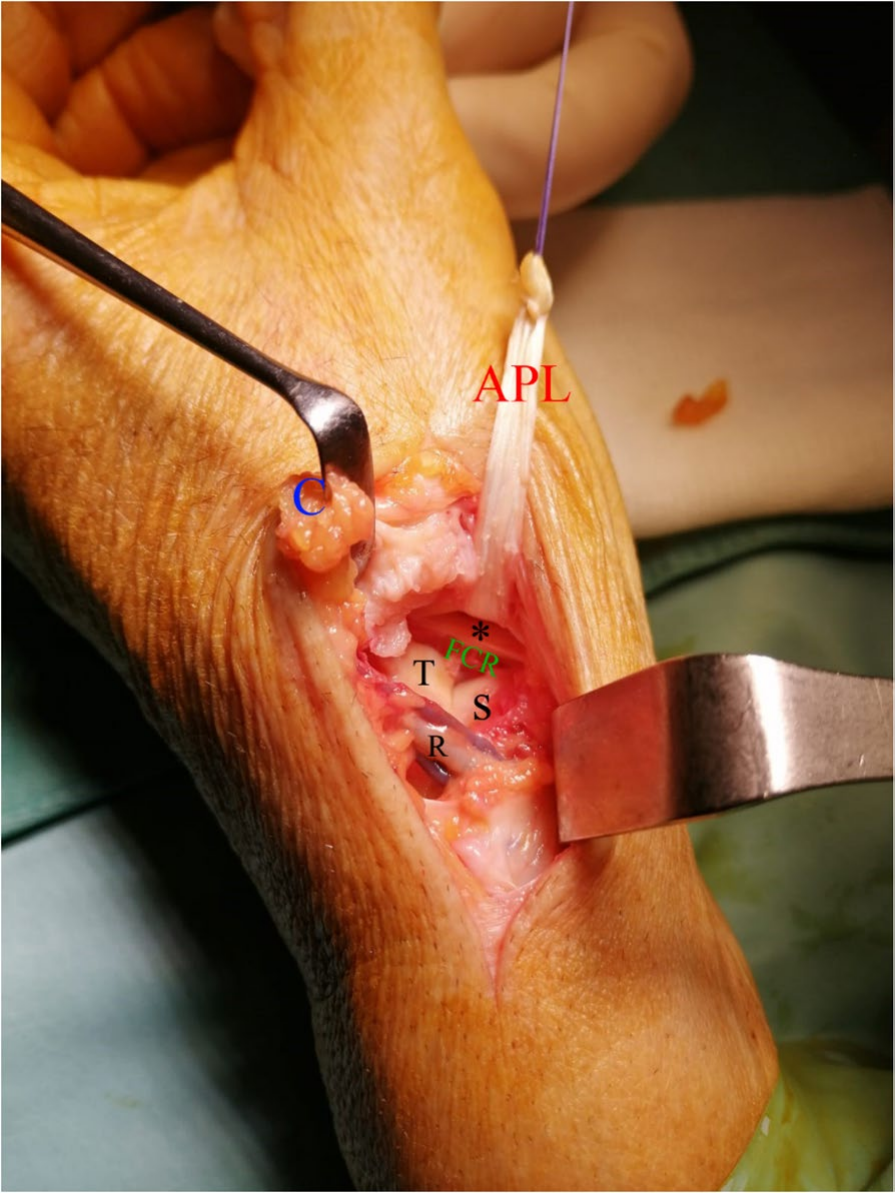
OLI technique. The radial artery (R) has been exposed. A “V-shaped" capsular flap (C) has been dissected and flipped distally under the retractor. The dorsal subunit of APL has been harvested and passed through the FCR. The symbol * indicated the split into the FCR. T: trapezoid. S: scaphoid (distal pole)

Now the technique is continued in two different ways:

- OLA: the APL subunit is looped around the FCR from palmar to dorsal at the bottom of the surgical field.
- OLI: the APL subunit is looped inside the FCR from palmar to dorsal at the bottom of the surgical field perforating the tendon with a curved and blunt instrument (Fig. 2).

Regardless of the variant (OLA or OLI), APL is anchored on the capsule through a hole in the triangular capsular flap using a nonresorbable woven 2.0 suture. The suture is performed to give stability to the first metacarpal bone, by exerting adequate traction on the tendon, to ensure sufficient hold of the suspension, with the first metacarpal bone in a neutral position [15]. Hemostasis is performed. Capsule is carefully closed, with no drainage. Skin exposure is sutured.

A plaster splint is made, to block the wrist, with thumb in anteposition and abduction, and free thumb IP joint, to be maintained for four weeks.

### Standard post-operative treatment

Any patient, who has a normal course that meets our expectations, gradually resumes his or her usual activities two months after surgery, with full recovery from the sixth month post-surgery.

Pain is managed by pain killer therapy with NSAIDs, based on the needs of the individual patient. After surgery, patients underwent a month of physical therapy with a physical therapist specializing in the hand. We consider one month of physiotherapy assisted by the specialized physiotherapist as the standard to achieve a good recovery of hand function for all patients. At the end of the month, the therapist decides in agreement with the patient whether it is appropriate to continue physiotherapy, depending on the functional recovery achieved.

However, the need for physiotherapy lasting more than one month is considered not attributable to a normal post-operative course, even if the result obtained is very good. For this reason, in our opinion, the need for further physiotherapy is to be considered as a complication.

Flexor carpi radialis (FCR) tendinitis is characterized by swelling and pain of volar radial wrist and is a frequent complication of this type of arthroplasty. This complication appears soon after plaster removal and is described by several patients as disturbing and life-dampening. Flexor carpi radialis (FCR) tendinitis could be resolved spontaneously, with splints and drugs, with spontaneous rupture of the FCR tendon or with surgical release of the tendon [15, 19].

#### Pre and post-operative assessments

Each patient was assessed pre-operatively and post-operatively at 3-month, 6-month and 2-year follow-ups at our outpatient clinic. Pain was investigated using a visual analogue scale (VAS), where zero indicates no pain and 10 the maximum unbearable pain, and overall satisfaction using a ten-point scale, where zero is totally dissatisfied and 10 is completely satisfied. Impact of disability was assessed with the Quick DASH questionnaire and MHQ questionnaire. The range of opposition was classified with the Kapandji score. Grip strength, tip and lateral pinch strength by using a dynamometer.

### Outcomes

The primary outcome was to assess and compare surgical outcomes comparing the two groups, in terms of subjective and objective evaluation for both groups at the last follow-up. The secondary outcome was to analyze any significant difference in terms of pain, DASH and MHQ score during the post-operative recovery, at 3 months (T1) and 6 months (T2) after surgery, and to outline any difference in complication rates.

#### Statistical analysis

All data were analyzed using IBM SPSS Statistics for Macintosh, Version 26.0 (IBM Corp., Armonk, N.Y.). For numerical data, mean ± SD were assessed.

The Wilk-Shapiro test was used to check for normal distribution for continuous variables.

The *t* test was used for parametric data. The Wilcoxon test for two dependent continuous non-parametric variables, the Mann–Whitney U-test for two independent continuous was used for non-parametric variables. For nominal data, the Chi-square test was applied to evaluate the *p* values associated. Statistical significance was expressed as *p* < 0.05.

## Results

Among 201 consecutive patients, 68 were identified by applying the inclusion and exclusion criteria. Group A (OLA) consisted of 35 patients (4 men and 31 women), with an average age of 64.4 (± 4.3) years. Group B (OLI) consisted of 33 patients (3 men and 30 women), with an average age of 62.8 (± 4.9) years.

Clinical and demographical features of participants are resumed in Table 1.

**Table 1:** Demographic and clinical features of studied patient *SD* Standard deviation, *WC* With collar, *BC* Blue collar, *CCI* Charlson Comorbidity Index

Demographics		Group A (OLA)	Group B (OLI)
**Number of patient**		**35 (51.5%)**	**33 (48.5%)**
**Age (years)** **(mean ± SD)**		**64.4 (± 4.3)**	**62.8 (± 4.9)**
**Gender**		**31 F, 4 M**	**30 F, 3 M**
**Occupation**	**WC**	5 (14.3%)	5 (15.2%)
	**BC**	30 (85.7%)	28 (84.8%)
**Comorbidity**	**Smokers**	6 (17.1%)	4 (12.1%)
	**Diabetes**	4 (11.4%)	5 (15.2%)
	**CCI**	2.3 (± 1.0)	2.2 (± 0.7)
**Eaton and Littler disease stage**	**Stage III**	26 (74.3%)	24 (69.7%)
	**Stage IV**	9 (25.7%)	9 (27.3%)
**The average duration of the symptoms (years)**		4.8 (± 1.7)	4.3 (± 2.1)
**Dominant side involved**		7 (33.3%)	6 (30.0%)
**Follow-up (years)** **(mean ± SD)**		2.1 ± 0.5	2.3 ± 0.6

About the primary outcome: no differences have been revealed in terms of clinical and functional results comparing the two techniques. At the last follow-up, patients obtained excellent results in both groups (*p* > 0.05), as resumed in Table 2.

**Table 2:** The results of preoperative (T0) and at the last follow up (two years, T3), expressed as mean ± SD

**Outcome**		**T0**	**T3**
**VAS pain score**	*Group A*	7.1 ± 1.0	0.1 ± 0.3
	*Group B*	7.3 ± 1.1	0.2 ± 0.4
	*p value*	0.36	0.63
**DASH questionnaire**	*Group A*	75.3 ± 36.0	16.2 ± 5.4
	*Group B*	76.2 ± 35.9	17.0 ± 6.3
	*p value*	0.95	0 .96
**MHO questionnaire**	*Group A*	24.3 ± 3.8	89.1 ± 4.2
	*Group B*	24.9 ± 4.9	89.5 ± 4.4
	*p value*	0.83	0 .58
**Kapandji score**	*Group A*	6.8 ± 0.9	9.0 ± 0.4
	*Group B*	6.6 ± 0.8	8.8 ± 0.4
	*p value*	0.41	0.42
**Tip pinch strength (Kg)**	*Group A*	2.6 ± 1.3	4.7 ± 0.5
	*Group B*	2.6 ± 1.3	4.6 ± 0.5
	*p value*	0.87	0.87
**Lateral pinch strength (Kg)**	*Group A*	3.7 ± 1.0	5.4 ± 0.5
	*Group B*	3.3 ± 1.1	5.4 ± 0.6
	*p value*	0.26	0.33
**Grip strength (Kg)**	*Group A*	12.0 ± 1.1	17.6 ± 1.4
	*Group B*	11.8 ± 1.1	18.0 ± 1.5
	*p value*	0.72	0.31
**Patient’s satisfaction**	*Group A*		9.2 ± 0.9
	*Group B*		9.5 ± 0.8
	*p value*		0.15

**Fig. 3:**
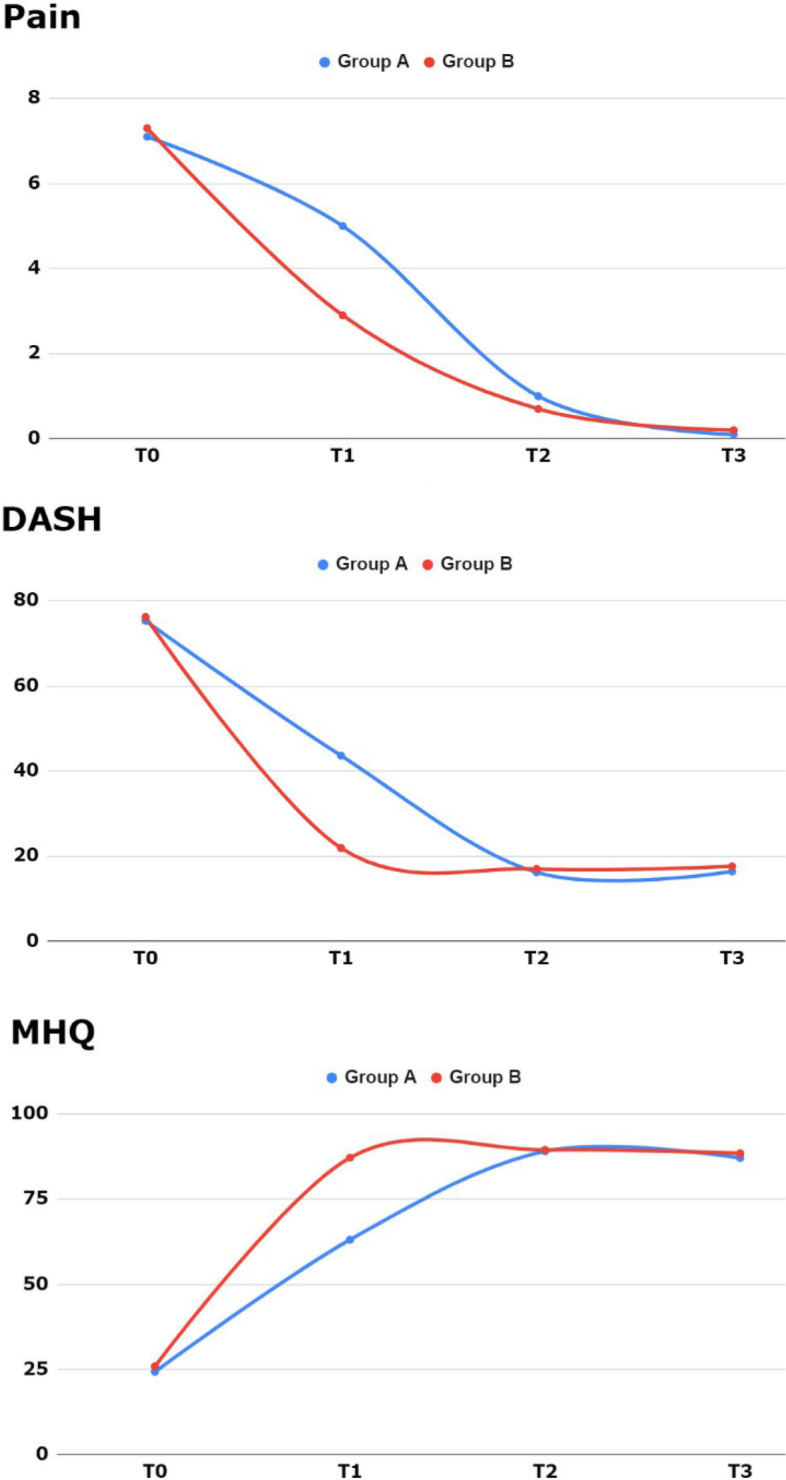
Progression of clinical improvement in terms of pain, MHQ and DASH scores pre-surgery (T0), after 3 months (T1), after 6 months (T2) and at the last follow-up (two years, T3)

However, in regards to the secondary outcome, the progression of clinical improvement was different between the two groups **(**Fig. 3).

Significant differences have been revealed in terms of pain, MHQ and DASH scores only after 3 months (T1), with better results with OLI technique. This clinical difference was not found to be statistically significant at further follow-ups (Table 3).

**Table 3:** Progression of pain, DASH and MHO outcomes pre-surgery (T0), after 3 months (T1), after 6 months (T2) and at the last follow-up (two years, T3). Results are expressed as mean ± SD

**Outcome**		**T0**	**T1**	**T2**	**T3**
**VAS pain score**	*Group A*	7.1 ± 1.0	5.0 ± 1.0	1.0 ± 0.6	0.1 ± 0.3
	*Group B*	7.3 ± 1.1	2.9 ± 0.7	0.7 ± 0.7	0.2 ± 0.4
	*p value*	.36	**< .00001**	.12	.64
**DASH questionnaire**	*Group A*	75.3 ± 36.0	43.6 ± 21.9	16.2 ± 5.4	16.4 ± 5.9
	*Group B*	76.2 ± 35.9	21.9 ± 9.0	17.0 ± 6.3	17.6 ± 7.1
	*p value*	.95	**.04**	.96	.95
**MHO questionnaire**	*Group A*	24.3 ± 3.8	63.1 ± 10.3	89.1 ± 4.2	87.1 ± 4.2
	*Group B*	24.9 ± 4.9	87.2 ± 7.5	89.5 ± 4.4	88.5 ± 7.4
	*p value*	.83	**< .00001**	.89	.78

TMC subluxation, subsidence or loss in suspension were checked through X-rays at the last follow-up. In accordance with Andrew J. Miller [20] et al. using the three methods described and relating our value of trapezial spaces to the average trapezial space ratio described. In no case was the our value lower than average ratio. Therfore TMC subsidence or loss in suspension were not found in patients at final follow-up. Only 1 patient of group A complained of complex regional pain syndrome (that has completely recovered in the first year after surgery), and scar hypersensitivity was reported by 5 patients in group A and 4 in group B, all of which completely recovered within 6 months. The number of patients who experienced FCR tendinitis was significantly higher in group A (21/35) than in group B (9/33) (*p* = 0.01). Almost half of the patients in group A underwent postoperative physiotherapy (16/35) compared to an eighth (4/33) of patients in group B (*p* = 0.006). No one underwent reoperation.

## Discussion

Aim of the surgical treatment of thumb carpometacarpal osteoarthritis is to eliminate pain, to provide stabilization and to standardize the modalities of surgical treatment [2, 4, 21–28].

Simple trapezial excision, initially performed in 1948 [5], is commonly complicated by shortening, instability of the metacarpal bone and important loss of strength [26].

Multiple procedures of trapezial excision with soft tissue arthroplasty were described and are distinguished between trapeziectomy + LRTI and Hemi-resection interposition arthroplasty.

Hemi-resection interposition arthroplasty provides similar benefits to trapeziectomy + LRTI in cases with no or mild joint subluxation and can be considered in younger, active patients [28].

LRTI is the most frequently surgical treatment performed for thumb carpometacarpal osteoarthritis and one option for a failed hemi-resection interposition arthroplasty or failed total joint prosthesis replacement. The most common complications are metacarpal subsidence and FCR tendinitis [2, 19, 20].

Different techniques are often performed, but limited comparative evidence exists between these techniques. The interposition material and suspension tenoplasty are considered essential surgical procedures to give more support to the thumb and to reduce the metacarpal subsidence.

Many anchor passages and many sutures are commonly performed to reinforce the suspension but there are no precise references regarding tension and filling in suspension teno-arthroplasty because is difficult to prove [2, 13, 14].

Catalano F. simplified the technique performing a single loop thinking that this procedure, together with careful capsuloplasty hourglass-like and intra-capsular fibrosis that occur in the 4 weeks of immobilization, was enough to stabilize the joint and to reinforce the suspension.

These modifications produced a reduction of surgical exposure because the tendon to be prepared is much smaller. A reduction of surgical times guaranteeing excellent results in terms of pain reduction, maintenance of strength and global function. Compared to a simple trapeziectomy, the modified technique promotes rapid functional recovery with excellent patient satisfaction. [2, 13, 14]

In this work, we perform a comparison between trapeziectomy + LRTI/OLA and trapeziectomy + LRTI/OLI. The risk of adhesion in both OLA and OLI LRTI is lower than in other LRTI because the incision and dissection is shorter resulting in a hemi-resection of the APL and due to there being only one loop rather than Ceruso’s double loop technique.

No differences have been revealed in terms of clinical and functional results comparing the two techniques. We did not find any metacarpal subsidence at the final follow-up.

However, significant differences have been revealed in terms of pain, MHQ and DASH scores with better results with OLI technique due to the lower incidence of FCR tendinitis.

The common techniques of LRTI could cause FCR tendinitis by overload on synovial of the FCR tendon [19]. OLI technique, compared to OLA and the other techniques described, in our opinion, leads to a lower risk of FCR tendinitis because the intratendinous passage does not cause rubbing on the synovial of the FCR tendon and allows the tendon loop to settle through spontaneous enlargement of the FCR tendon fibers.

In the case of FCR tendinitis, a spontaneous resolution often occurs in a few months. Corticosteroid injection or physiotherapy is rarely needed.

There have been no ruptures of the FCR tendon, but if it occurs after the end of the fibrosis stabilization, we think that the metacarpal subsidence is not a complication to be considered and no treatments are necessary. However, a longer follow-up might lead to different findings.

## Conclusions

The further modification made to Catalano’s technique maintains the improvement of the original technique and represents a further improvement in post-operative management.

## Data Availability

The data used to support the findings of this study are available from the corresponding author upon request.

## Abbreviations

TMC: Trapeziometacarpal
APL: Abductor pollicis longus
FCR: Flexor carpi radialis
OLA: Once Looped Around
OLI: Once Looped Inside
LRTI: Ligament Reconstruction and Tendon Interposition
MP: Metacarpo-phalangeal
CCI: Charlson Comorbidity Index
MHQ: Michigan Hand Outcome
VAS: Visual analogue scale

## References

[1] Haara MM, Heliövaara M, Kröger H (2004). Osteoarthritis in the carpometacarpal joint of the thumb. Prevalence and associations with disability and mortality. J Bone Joint Surg Am..

[2] Taccardo G, DE Vitis R, Parrone G, Milano G, Fanfani F (2013). Surgical treatment of trapeziometacarpal joint osteoarthritis. Joints.

[3] Dell PC, Brushart TM, Smith RJ (1978). Treatment of trapeziometacarpal arthritis: results of resection arthroplasty. J Hand Surg Am.

[4] Eaton RG, Littler JW (1973). Ligament reconstruction for the painful thumb carpometacarpal joint. J Bone Joint Surg Am.

[5] Gervis WH (1948). Excision of the trapezium for osteoarthritis of the trapezio-metacarpal joint. Postgrad Med J.

[6] Burton RI, Pellegrini VDJ (1986). Surgical management of basal joint arthritis of the thumb. Part II. Ligament reconstruction with tendon interposition arthroplasty. J Hand Surg Am..

[7] Muller GM (1949). Arthrodesis of the trapezio-metacarpal joint for osteoarthritis. J Bone Joint Surg Br.

[8] Lemoine S, Wavreille G, Alnot JY, Fontaine C, Chantelot C (2009). Second generation GUEPAR total arthroplasty of the thumb basal joint: 50 months follow-up in 84 cases. Orthop Traumatol Surg Res.

[9] Yuan F, Aliu O, Chung KC, Mahmoudi E (2017). Evidence-based practice in the surgical treatment of thumb carpometacarpal joint arthritis. J Hand Surg Am.

[10] De Maio F, Farsetti P, Potenza V (2019). Surgical treatment of primary trapezio-metacarpal osteoarthritis by trapeziectomy and ligament reconstruction without tendon interposition. Long-term results of 50 cases. J Orthop Traumatol Off J Ital Soc Orthop Traumatol.

[11] Mathoulin C, Moreel P, Costa R, Wilson SM (2008). Abductor pollicis longus ‘hammock’ ligamentoplasty for treatment of first carpometacarpal arthritis. J Hand Surg Eur.

[12] Weilby A (1988). Tendon interposition arthroplast of the first carpo-metacarpal joint. J Hand Surg Br Eur.

[13] Ceruso M, Delcroix L (1995). Artroplastica tendinea in sospensione nel trattamento della rizoartrosi. Revisione di 80 casi operati. Atti XXXIII Congresso S.I.C.M.

[14] Ceruso M, Innocenti M, Angeloni R, Lauri G, Bufalini C (1991). L’artrosi del primo raggio digitale. Riv Chir Mano.

[15] Berto G, Pegoli L, Cortese P (2010). La nostra esperienza nel trattamento della rizoartrosi: studio su 792 casi consecutivi trattati con artroplastica in sospensione. Riv Chir Mano.

[16] Singer MS, Kandel WA (2016). Slip abductor pollicis longus suspension tendinoplasty for management of trapezio-metacarpal joint osteoarthritis. Int Orthop.

[17] Passiatore M, De Vitis R, Cilli V (2021). The Italian version of the Michigan Hand Outcomes Questionnaire (MHQ): translation, cross-cultural adaptation and validation. J Hand Surg Asian Pac.

[18] Padua R, Padua L, Ceccarelli E (2003). Italian version of the Disability of the Arm, Shoulder and Hand (DASH) questionnaire. Cross-cultural adaptation and validation. J Hand Surg Br.

[19] Low TH, Hales PF (2014). High incidence and treatment of flexor carpi radialis tendinitis after trapeziectomy and abductor pollicis longus suspensionplasty for basal joint arthritis. J Hand Surg Eur.

[20] Miller AJ, Jones CM, Martin DP, Liss FE, Abboudi J, Kirkpatrick WH, Beredjiklian PK (2018). Reliability of metacarpal subsidence measurements after thumb carpometacarpal joint arthroplasty. J Hand Microsurg.

[21] Allieu Y (2021). Anatomically based radiological classification of thumb basal joint arthritis. Hand Surg Rehabil.

[22] Wajon A, Ada L, Edmunds I. Surgery for thumb (trapeziometacarpal joint) osteoarthritis. Cochrane Database Syst Rev. 2005;(4):CD004631. 10.1002/14651858.CD004631.pub2. Update in: Cochrane Database Syst Rev. 2009;(4):CD004631. PMID: 16235371. 10.1002/14651858.CD004631.pub2 16235371

[23] Laronde P, Duriez P, Oca V, d'Almeida MA, Hustin C (2022). Thumb basal joint arthritis: new classification, diagnostic and therapeutic algorithm. Hand Surg Rehabil.

[24] Bonetti MA, Rovere G, Fulchignoni C (2020). Autologous fat transplantation for the treatment of trapeziometacarpal joint osteoarthritis. Orthop Rev (Pavia).

[25] van Laarhoven CMCA, Schrier VJMM, van Heijl M, Schuurman AH (2019). Arthrodesis of the carpometacarpal thumb joint for osteoarthritis; long-term results using patient-reported outcome measurements. J Wrist Surg.

[26] Saab M, Chick G (2021). Trapeziectomy for trapeziometacarpal osteoarthritis. Bone Jt Open.

[27] Holme TJ, Karbowiak M, Clements J (2021). Thumb CMCJ prosthetic total joint replacement: a systematic review. EFORT Open Rev.

[28] Spiteri M, Giele H (2020). Systematic Review of Thumb Carpometacarpal Joint Hemiresection Interposition Arthroplasty Materials.

